# Cell-Biomaterial Mechanical Interaction in the Framework of Tissue Engineering: Insights, Computational Modeling and Perspectives

**DOI:** 10.3390/ijms12118217

**Published:** 2011-11-21

**Authors:** Jose A. Sanz-Herrera, Esther Reina-Romo

**Affiliations:** School of Engineering, University of Seville, Camino de los descubrimientos s/n, 41092 Seville, Spain; E-Mail: erreina@us.es

**Keywords:** cell mechanics, adherent cells, cell-biomaterial interaction, tissue engineering, computational modeling

## Abstract

Tissue engineering is an emerging field of research which combines the use of cell-seeded biomaterials both *in vitro* and/or *in vivo* with the aim of promoting new tissue formation or regeneration. In this context, how cells colonize and interact with the biomaterial is critical in order to get a functional tissue engineering product. Cell-biomaterial interaction is referred to here as the phenomenon involved in adherent cells attachment to the biomaterial surface, and their related cell functions such as growth, differentiation, migration or apoptosis. This process is inherently complex in nature involving many physico-chemical events which take place at different scales ranging from molecular to cell body (organelle) levels. Moreover, it has been demonstrated that the mechanical environment at the cell-biomaterial location may play an important role in the subsequent cell function, which remains to be elucidated. In this paper, the state-of-the-art research in the physics and mechanics of cell-biomaterial interaction is reviewed with an emphasis on focal adhesions. The paper is focused on the different models developed at different scales available to simulate certain features of cell-biomaterial interaction. A proper understanding of cell-biomaterial interaction, as well as the development of predictive models in this sense, may add some light in tissue engineering and regenerative medicine fields.

## 1. Introduction

Manipulation of cells to seed them on artificially engineered matrices is currently a common procedure in the field of tissue engineering. The interaction of cells on these matrices, which replaces the biological extracellular matrix (ECM), is critical in order to get a functional tissue engineering product which allows cultured cells to develop their normal activity. Some of the most important phenomena are experimentally observed, when cells are placed on a certain biomaterial regard to mechanical factors. Therefore, a concise understanding of cell-biomaterial interaction includes a deep understanding of cell biology and cell-extracellular matrix interaction, to be extrapolated to the biomaterial surface for tissue engineering applications.

In this section, the main functions and structures of the cell, as wells as the most interesting mechanical phenomena observed in the lab regarding to cell-biomaterial interaction, are reviewed. Furthermore, a brief introduction to the field of tissue engineering is provided in this section. The rest of the paper is focused on cell-biomaterial interaction with an emphasis on the available computational methods and new trends in this concern, with the aim of predicting some interesting observed phenomena. The computational approach, or *in silico* approach, of cell-biomaterial interaction problems may be an important tool in the design of functional tissue engineering products and biomaterials.

### 1.1. Structures and Functions of Living Cells

The cell is a unique biological living material composed of different hierarchical structures at different spatial levels. At the nanometer scale, we can distinguish between four great classes of macromolecules which build the living organisms and the different structures of the cells, namely, proteins, carbohydrates, lipids and nucleic acids.

From a structural perspective, the anatomy of eukaryotic cells is divided into plasma membrane, cytoplasm and nucleus. Plasma membrane is a lipid bilayer structure composed of intermembrane proteins, cholesterol and glycoproteins. It is a barrier or boundary of the cell which separates intracellular structures and fluids with extracellular ones. The plasma membrane plays a dynamic role in cellular activity and its main functions are transport, enzymatic activity, signal transduction, intercellular joining and cell-cell recognition. Especially important is the role that the plasma membrane plays in cell attachment to the extracellular matrix (ECM) or artificial biomaterial. In view of this importance, it is reviewed in depth in section 2.1. On the other hand, the cytoplasm contains organelles being them the metabolic machinery of the cell. Organelles are different structures within the cell which develop the necessary biochemical and also mechanical functions for life. For further reading on cell’s organelles, the reader is referred to [1–3]. Finally, the nucleus is a double lipid bilayer (barrier) which comprises the DNA and other important molecules. The nucleus is considered the control center of the cell and it regulates gene expression.

Cells, as living organisms, are continuously involved in a dynamical process of biochemical signaling and functions development, which are biologically featured by certain cell behaviors such as growth, differentiation, migration or apoptosis. Some of these processes are somehow regulated or explained by mechanical cues. This issue is discussed next with an emphasis on the mechanical characteristics of cell-biomaterial interaction.

### 1.2. Mechanical Aspects of Cell-Biomaterial Interaction

The main mechanical component of the cell is the cytoskeleton (CSK). It is a load-bearing structure which transmits and sustains forces exerted by the cell at different locations. CSK is considered the cell skeleton or scaffolding of the cell and is composed of microtubules, actin filaments and intermediate filaments. Briefly, microtubules are rigid, long straight, dynamically unstable hollow tubes (heterodimers) arranged by the centrosome complex. Microtubules maintain cell shape by resisting compression and their functions are motility via flagella or cilia and movement of chromosomes during division as well as organelles. Actin filaments, usually called microfilaments, are 6–8 nm width double helix structures which resist tension, also generate tension through the actin-myosin complex, *i.e.*, actomyosin fibers, provide mechanical support to cells, and hardwire the cytoplasm with the surroundings to support signal transduction; and allow cell motility and cell division. Finally, intermediate filaments are formed by a large and heterogeneous group of proteins and their functions are to resist tension, anchoring nucleus and some other organelles, providing mechanical stability to cells and define some inter-cellular unions (see [4,5] for a further reading).

CSK is involved in the process of cell attachment to the ECM through the plasma membrane. Therefore, a proper understanding of the plasma membrane (linked to the CSK) and the ECM interaction may add some light in the design of functional artificial substrate to promote cell function and finally the formation of engineered tissues. This important issue is discussed in section 2. However, there are some other important cell junctions which are less important for biomaterial interaction but are critical for the formation of tissues, *i.e.*, cell-cell adhesions. Cells can attach to each other by the establishment of four different unions: desmosome, tight junctions, gap junction and microvilli. Cell-cell interaction is a key point in tissue engineering as well, which is out of the scope of this paper. The reader is referred to [3] for a further reading.

In recent years, it has been evidenced that cell interaction with artificially engineered substrates and subsequent cell behavior is strongly influenced by mechanical factors. In this sense, it has been shown that cells exhibit very different behaviors depending on the elasticity of the substrate they are anchored to, or equivalently, on the elasticity of the extracellular microenvironment [6]. Examples of this corroboration are the fact that cancer cells grow on soft agar rather than on a solid substrate [7], or the differentiation of different cell phenotypes modulated by substrate stiffness. Engler *et al.* [8] demonstrated that mesenchymal stem (undifferentiated) cells are able to differentiate to neuron-like cells when placed on a substrate with stiffness of the order of 1 kPa, or differentiate to myoblasts-like cells when the substrate stiffness is around 10–20 kPa. These results may have important implications for tissue engineered substrates, although a detailed description of the related physical and molecular mechanisms are far to be elucidated. One key point to unravel the intricacies of the behavior of cells on different substrates (regarding to stiffness) may be the analysis of adhesion cell points to the substrate, *i.e.*, focal adhesions. It is shown that cells respond to the resistance of the substrate, by adjusting its adhesions, cytoskeleton, and overall state [6]. In fact, soft, lightly, cross-linked gels with elasticity of the order of 1 kPa show diffuse dynamic adhesion complexes, whereas stiff, highly cross-linked gels with elasticity ranging from 30 to 100 kPa show cells with stable focal adhesions [6]. Moreover, inhibition of actomyosin (contractility motor) eliminates focal adhesions, and in contrast contractility stimulation drives integrin (an adhesion-mediated protein) aggregation into adhesions [9–11]. Leader *et al.* [12] had already reported on a positive correlation between cell adhesion and cell contractility. A simple theory to explain these results, linked to the observed behavior of cells on different substrates, may be stated as follows. Considering matrix strains (the relationship between stress and stiffness) as a constant, it is then clear that cells on soft substrates need be less contractile than on stiff cells and hence adhesions need not be as strong [6]. This explanation is in connection with measured adhesion forces on cells on soft and rigid substrates [13] and also with the observed evidence that neurons apply very little stress to substrates and then they prefer soft gels [14].

Overall, the effect of substrate stiffness on cell is phenomenologically featured by a certain cell response such as migration. The phenomenon of migration is the coordinated action of cell movement in a four-step based motion pattern: protrusion of the leading edge, formation of new adhesions near the front, cell contraction and release of the real adhesions [15,16]. Specifically, reports have demonstrated a dependence of migration velocity on substrate stiffness. Research with epithelial cells [9], fibroblasts [17] and smooth muscle cells [18,19] have shown that cells migrate from softer regions to stiffer ones when subjected to a gradient in substrate stiffness. The velocity is maximal for a certain intermediate stiffness after which it decreases. This phenomenon is termed *durotaxis*. On the other hand, *tensotaxis* is referred to as the ability of cells to crawl to areas of strained substrates [20]. This phenomenon was experimentally evidenced by Riveline *et al.* [21]. These authors seeded fibroblasts on flexible substrate and applied force just by pressing the cell using a micropipette. Results showed an increasing concentration of focal adhesions at the site of the applied force. Similar results were obtained by Tamada *et al.* [22], concluding that stretching may create binding sites for activator and adapter proteins and thus alter the balance between cell protrusion and contractility. As in previous observed cell behaviors guided by mechanical cues, the phenomenon of tensotaxis is not yet clearly understood. It may be explained by the fact that stretching of the cell may cause conformational changes to uncover substrate binding site since it is known that talin (a key adhesion-mediated protein) must unfold to bind vinculin (adhesion-mediated protein) [23,24].

### 1.3. Tissue Engineering Science

Tissue engineering antecedents date form late 80s and beginning of the 90s with the interest of physicians to overcome many complications of the available techniques for the treatment and repair of damaged tissues. Among these techniques one can cite tissue/organ transplantations or tissue grafting. Both organ transplantation and tissue grafting remain imperfect solutions [25] and in this context the so-called field Tissue Engineering emerged, with the aim of creating substitutes which replace the damaged tissue or organ function.

Tissue engineering is an interdisciplinary field that applies the principles of engineering and the life sciences towards the development of biological substitutes that restore, maintain, or improve organ or tissue function [26]. The interdisciplinarity of this field includes Biology, Materials Science and Engineering as the main roots.

There exist different approaches for organ/tissue repairing or regeneration according to the specific organ/tissue features. Nevertheless, the tissue engineering methodology follows a similar generic scheme:

- *Cell recruitment: isolation and expansion*. Tissues and organs are made of cells and the specific function of a tissue or organ can only be developed by those cells. Potential limitation of cell therapy is bounded by restricted cell niches. For this, adult stem cells can be isolated and after expanded and differentiated *in vitro*. In order to improve tissue regeneration, signal molecules, such as growth factors, are used in combination with cell therapy;
- *Biomaterial interaction*. Recruited cells are usually seeded *in vitro* onto artificial biomaterial matrices, *i.e.*, scaffolds. The aim of these matrices is to give support to the cells to develop its specific function during the healing or regenerative process. Cell-biomaterial interaction is critical and the biomaterial should mimic the extracellular space that it replaces. This issue is the focus of this review;
- *Implantation.* Seeded matrices, performed *ex vivo*, are implanted *in vivo* for tissue repairing. Here, biocompatibility must be assured in order to prevent an immunological rejection. Moreover, the vascularization of the surrounded tissue should occur within the artificial matrix.

In the next section, the main components of cell-ECM interaction as well as the implicated mechanisms are reviewed. Also, some of the most important features of cell-biomaterial interaction in the framework of tissue engineering are considered in section 2.4.

## 2. Mechanisms for Cell-ECM and Artificial Substrate Interaction

Plasma membrane and ECM are the main biological structures involved in the process of cell attachment. These two components are briefly described in this section in order to provide below a detailed description of the process of cell-ECM interaction during adhesion. The proper understanding of the natural adhesion mechanisms provides insights to engineer artificial substrates which allow cell seeding and normal cell activity in tissue engineering applications. This issue is outlined at the end of this section.

### 2.1. Plasma Membrane

Plasma membrane is a surface membrane that defines the limits and the connectivity of the intracellular and extracellular regions. It is selectively permeable to ions and organic molecules, and controls the movement of substances in and out of cells [1].The main functions of the membrane are transport, enzymatic activity, signal transduction, intercellular joining, cell-cell recognition and CSK-ECM attachment. Plasma membrane architecture comprises an arranged phospholipid bilayer being a thin layer of organized amphipathic phospholipids such that the hydrophobic (water fearing) tail regions are away from the surrounding polar fluid, and the hydrophilic (water loving) head are in contact with cytosolic and extracellular domains. Other lipids contained in the membrane are glycolipids and cholesterol, which has a steroid ring structure, although the most abundant lipids are phospholipids. Other important components of the plasma membrane structure are integral proteins. These proteins are contained within the transmembrane region and support the selective movement of ions and small molecules from one side of the membrane to the other, sense a ligand on one side of the membrane and transmit a signal to the other side, and provide mechanical linkage for other proteins on either side of the membrane. The proteins that move materials across the membrane can be functionally divided into channels, pumps and transporters [27]. Among the different integral proteins, especially important for cell adhesions are cadherins, desmosomes and integrins. Cadherins and desmosomes are specialized proteins for cell-cell adhesions which ensure that forming tissues cells are bound together. On the other hand, integrins are adhesion-mediated proteins which link cells to fibronectin or laminin in the extracellular matrix [27].

### 2.2. ECM

**Figure 1:**
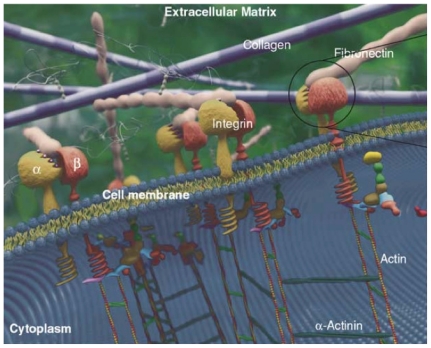
Structure and components of the extracellular matrix. From [29].

The ECM is the surrounding space between cells which provides support and anchoring, *i.e.*, adhesion. Other related functions of the ECM are to capture growth factor proteins, being also essential in biological processes such as growth and wound healing. ECM plays also an important role in diseases such as fibrosis or tumor invasion [1,28]. The main constituents of the ECM (see Figure 1) are proteoglycans, fibers and other molecules. Proteoglycans are long chain carbohydrate polymers negatively charged to attract water molecules via osmosis in order to keep the ECM hydrated. Furthermore, fibers include collagen and elastin. Collagen is a protein arranged in the ECM as a fibrillar structure which provides support to cells when attached. On the other hand, elastin provides elasticity and stretch capability to ECM, allowing deformation during cell motion and attachment. The rest of the proteins found in the ECM space are fibronectin and laminin which are involved in the process of cell-ECM adhesion. Specifically, fibronectin connects cells with collagen fibers in the ECM by binding them to cell membrane integrins, causing a reorganization of the cell’s cytoskeleton which facilitates cell movement through the ECM. Details of cell-ECM attachment are given in the next section.

### 2.3. Cell-ECM Interaction

There exist different kinds of structural cell linkages with the ECM. In one hand, cells can attach the basal lamina (lining outer surface of cell membrane) via integrins to the intermediate filaments of the CSK. On the other hand, cells are able to anchor proteins of the basement membrane of the ECM via integrins to the actin filaments of the CSK. The latter kind of junction is referred to as a hemidesmosome junction, whereas the former are focal adhesions [4]. Specifically, cells perform dynamically stable sites for adhesion, *i.e.*, focal adhesions, with the ECM during migration. In this process, integrins and related molecules migrate to the leading edge of lamellipodia or filopodia protrusion (front of the cell) which attach to the ECM [15]. The case in which cells are embedded within the matrix has not been considered in this manuscript.

**Figure 2:**
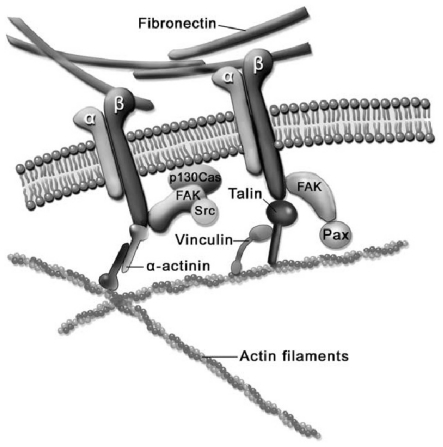
Scheme of the different intra-, trans- and extra-cellular proteins involved in adhesion of the cytoskeleton (CSK) to the extracellular matrix (ECM). From [36].

**Figure 3:**
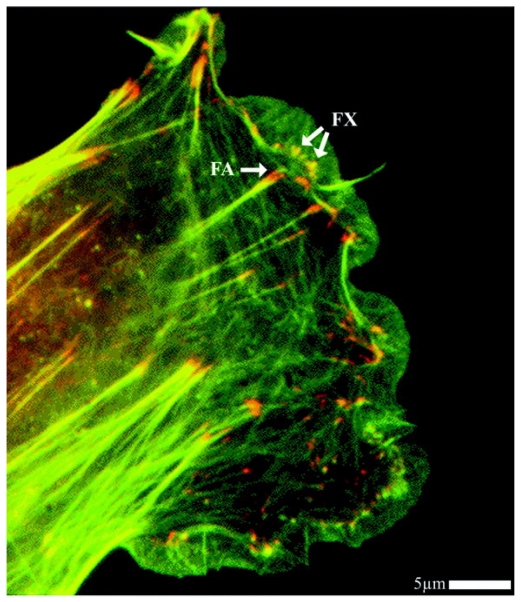
Representation of a fibroblast showing actin filaments in green (counterstained for F-actin with phalloidin), and focal complexes and focal adhesions in red (immuno-labelled for vinculin). From [41].

Focal adhesion is a multi-molecular complex connecting the ECM with the actin fibers of the CSK. Heterodimeric transmembrane integrin receptors bind matrix proteins via their ECM domains, while their cytoplasmic domains are associated with a dense submembrane plaque containing more than 50 different proteins including structural elements as well as signal transduction proteins such as FAK, Src, ILK, *etc.* [30,31]. Some of these proteins are very important for the establishment of focal adhesions. Specifically, talin, a cytoplasmic protein with a globular head and an elongated rod, is a key protein for cell attachment [32]. The globular head of talin binds to β-integrin [33] and F-actin [34], and contains 11 vinculin-binding sites [35], see the scheme depicted in Figure 2 [36]. Vinculin is a cytoplasmic protein that may function as a structural reinforcement. It consists of a globular head, a proline-rich neck region, and a rod-like tail domain, which contains binding sites for many other cytoplasmic proteins [37,38]. Vinculin however may not be present in the molecular mechanism of the focal adhesion since talin may bind directly to the actin bundling actinin (α-actinin) and then to the actin fiber (see Figure 2). In fact, cells with vinculin disruption, however, can still form focal adhesions, but display reduced ability to spread and increased cell motility [39]. Nevertheless, cells with disrupted talin function fail to form focal adhesions and exhibit spreading defects [40]. Finally, the exterior end of the integrin binds to fibronectin contained within the ECM space (see Figure 2). Focal adhesions first appear as a small, punctate adhesion at the cell edge, *i.e.*, focal complex, after which they grow in size and transform into a mature focal adhesion [30], see Figure 3.

It is clear that the exposed molecular mechanism of adhesion is a compound of mechanical cues which are transcribed into a cascade of biochemical signals [30]. It has been described within the generic term *mechanotransduction* [36]. In fact, focal adhesions have been proposed as a candidate for mechanotransduction [30,36,42]. Experimental evidence of the transduction of mechanical forces into a transcription of biochemical signals, finally leading to a certain cell function, such as motility, morphogenesis, proliferation or apoptosis, have been reported. Wang *et al.* [43] performed an experiment that applied mechanical stresses with a magnetic twisting device directly to cell surface receptors, showing that the extracellular matrix receptor, β-integrin, induced focal adhesion formation and supported a force-dependent stiffening response, whereas non-adhesion receptors did not. Other experiments support the hypothesis that application of forces to adhesion-mediated proteins increases the strength of the cytoskeletal anchorage [44,45].

On the other hand, it has been shown that focal adhesions become stable and grow under the application of external forces, such as application of direct tension to the cell [21] or to the flexible substrate [46].

### 2.4. Cell-Biomaterial Interaction on Artificial Substrates

Once a biomaterial is implanted in the human body, it produces a number of reactions by the surrounding tissues and cells, and the biomaterial’s interface. A measure of the tissue-biomaterial interaction is given by the biomaterial’s *biocompatibility*. For example, it is known that fibrous encapsulation occurs both in metals and polymeric constructs [47,48], which is featured by the decreasing adhesion of tissue-specific cells and the presence of a fluid-filled void between the tissue and implant [49]. Macrophages are the dominant cells in this process and regulate the reaction of the body to the foreign biomaterial. On the other hand, wettability is an important characteristic of the biomaterial in order to allow the early stages of cell adhesion onto the biomaterial surface [50]. Moreover, experiments performed *in vitro* suggest that endogenous proteins become rapidly adsorbed to the biomaterial surface driven by surface free energy [51,52] providing a structural framework on which cellular adhesion may initiate. In order to enhance the properties of biomaterials in the early stages of interaction with cells and tissues, surface and chemical modifications techniques are applied [53,54] yielding the third generation of biomaterials [55]. In addition, adherent cells need to be attached to the extracellular space (or replacing one) to fulfill their proper functions such as growth, differentiation or proliferation. Furthermore, this process is partially regulated by mechanical factors as previously discussed above. This is the focus of this review in the framework of artificial substrate interaction which is discussed next.

Engineered artificial constructs available for cell adhesion must mimic the extracellular space it replaces for a proper cell function and development. Due to the great level of detail of the natural ECM, exposed above, it makes this issue a challenging problem in *biofabrication*. A natural way to approach this problem is to use the natural ECM as a biologic scaffold material [56] or a combination of biofabricated scaffolds and the incorporation of biological materials. However, maintaining the quality and activity of harvested biopolymers throughout their extraction, processing, and remodeling is challenging, and some voices concern over the direct use of animal-derived material in a medical setting [57]. On the other hand, artificially fabricated biomaterials require a level of detail of nanometers in order to replicate the molecular processes involved in the phenomenon of cell-ECM adhesion. Nanotechnology processes, such as chemical vapor deposition, polymer phase separation, colloidal lithography, photolithography, and electron beam lithography (see [58] for a review) have been applied to provide the artificial biomaterial with topographical and chemical cues which react with the cell improving the process of adhesion. In particular, cells are able to respond to nanotopographical features showing diverse behaviors such as adhesion, cell orientation, cell motility, surface antigen display, cytoskeletal condensation, activation of tyrosine kinases, and modulation of intracellular signaling pathways that regulate transcriptional activity and gene expression [59,60]. Specifically, reports have shown perturbed changes on filopodial protrusions for a characteristic nanoroughness behind 70 nm [61]. On the other hand, chemical modification of the biomaterial by coating the surface with ligand that binds to specific extracellular receptors has been largely proven to enhance seeding efficiencies [62–65], and recently in references [53,54]. In particular, in these papers, fibronecting or RGD-peptides coatings (which are recognized as a binding site by several members of the integrin family) have been shown to be efficient in cell adhesion and proliferation. Moreover, fibronectin coating has demonstrated an increasing cell capability to build focal adhesions on an array of micropillars available for the measurement of forces exerted by the smooth muscle cells during adhesion [66].

These results suggest that a proper understanding of the molecular mechanisms involved in adhesion may yield the development of predictive models which allow one step forward in the biofabrication of functional advanced biomaterials in the framework of tissue engineering. This issue is reviewed in detail in the next section.

## 3. Cell-Biomaterial Interaction Models

Interest of cell-adhesion *in silico* provides a fundamental quantitative under standing of adhesion of cells to surfaces, either synthetic o or natural ECM, and can lead to understand ding *in vivo* phenomena, interpretation of *in vitro* assays, and analysis and design of biotechnological processes [67]. In addition, computational models can decouple different variables of an experiment to study their influence on the system and help to characterize general principles. Depending on the length scale under consideration there are several approaches for t the mathematical modeling of the cell-substrate interaction. At the atomistic scale, discrete models m make use of Molecular Dynamics (MD) to analyze the force response of several molecular structures. The behavior of molecular bonds may also be modeled at the mesoscopic scale with phenomenological rules that relate kinetics and m mechanics. Finally, the continuum models face this problem at a larger scale.

### 3.1. Molecular (Protein-Based) Models

**Figure 4:**
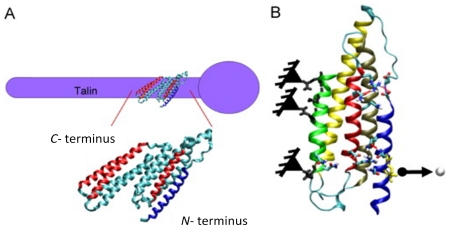
(**A**) Crystal structure of talin *n* ribbon representation and (**BB**) model setup for molecular dynamics simulation [35].

It is well known that proteins are involved in transmitting forces from the cell membrane to the actin cytoskeleton, as previously d discussed. The ligand-receptor (L-R) interaction is the unit physical linkage between a cell and the EC CM at the atomistic/molecular scale and its m modeling is the challenge of the protein based models. These models simulate the mechanics of a protein (or protein groups) based on molecular dynamics. In particular, steered MD (SMD) enables computational modeling of molecular structures unbinding at the atomistic level by applying an external force [68]. This allows characterizing L-R involved in the EC-substrate contacts. SMD has been very u useful in the study of the fibronectin protein, which binds the ECM to the integrins [69–71]]. The ligand-receptor biotin-streptadivin has also been well characterized using SMD [68,72]. Streptavidin-biotin is the “anchor” that stabilizes the initial EC-substrate contact, bringing the cell membrane close to adsorbed fibronectin. In other studies, Lee *et al.* [35] and Hytönen & Vogel [73] have performed an MD analysis of a key mechanosensing protein (talin) in focal adhesion (see Figure 4). Golji and Mofrad [74] and Golji *et al*. [75] examined, using MD simulations, the stretching and activation of vinculin, a protein that also plays a critical role in focal adhesion and activation.

Despite the valuable information obtained from MD simulations, these models require an extraordinary amount of computational power. In fact, it is not currently computational feasible to model an entire focal adhesion complex at the atomistic level [68].

### 3.2. Kinetics and Chemomechanical Adhesion Models

**Figure 5:**
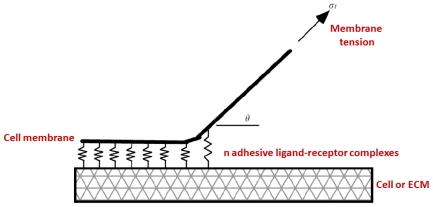
Model of cell adhesion that combines kinetics and mechanics. The L-R bonds are modeled as continuously distributed springs linking the two surfaces (adapted from [79]).

A general idea to estimate the interactions between the ligand-receptor bonds in cell adhesion is to relate the kinetic rates or binding affinities and the mechanical variables. As illustrated in Figure 5, these models combine two engineering science disciplines: the mechanics of peeling of an adhesive tape [76,77] and the thermodynamics of L-R binding, which account for the kinetic energy of the adhesive bonds [78]. The different models proposed depend on the kinetic rules for binding and unbinding and the force applied.

The first theory for describing the non-equilibrium dissociation of adhesion bonds under constant force was established by Bell [80] in a thermodynamic framework. He introduced the force dependence on the rupture kinetics of biomolecular bonds and assumed an exponential increase of the dissociation rate with force [80]:

(1) $$k_{off}(F)=k_{0}e^{F/F_{B}}$$

with *k_off_* (*F*) being the dissociation rate (off-rate) under force, *k_0_* the spontaneous off-rate in the absence of force and *F_B_* a parameter.

This deterministic model has been extended further and applied to study more specific problems. For instance, the peeling of a deformable one-dimensional membrane [81] or the rolling of leucocytes in shear flow [67] has been solved numerically to extract a critical tension or shear rate for rupture. In a later study, Seifert *et al*. [82,83] analyzed the dynamic behavior of a molecular bond cluster subjected to linearly ramping forces.

**Figure 6:**
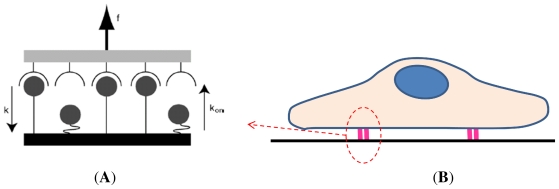
(**A**) Schematic representation of an adhesion cluster under force of 5 parallel adhesion bonds; (**B**) Scheme of a c cell adhering to a substrate through two s sites of adhesion. Adapted from [94].

However, these first mechanical and thermodynamical models [67,78,80,82,83] are deterministic and highly idealized, complicated and based on nontrivial hypothesis [79]. In contrast to deterministic models, a probabilistic framework might be more realistic due to the stochastic nature of the chemistry of L-R binding, which becomes especially significant when the number of reacting molecules is relatively small as is the case with h cell surface receptor molecules [84,85]. Probabilistic models treat stochastic kinetics either with master equations [65,84,86–89] or via Monte Carlo simulations [90–92]. Cozens-Roberts *et al.* [65] emphasized the limits of previous deterministic models in situations where cells might be bound by a few molecules. With a stochastic version of the Bell model, they provided a probabilistic model that yielded different predictions concerning the time required for cell attachment and detachment. Later, Erdmann and Schwarz [87–89] proposed a more rigorous theory of cluster lifetime based on the one-step m master equation in stochastic dynamics. They studied the adhesion cluster stability under mechanical load, both for constant force [87,88] and linearly rising force [89] and included the effect of receptor-ligand distance [93] (Figure 6). These m models demonstrate that force might regulate the internal state of focal adhesion. Introducing a two-spring model, they also showed in a quantitative way how internal dynamics can in principle be c coupled to extracellular elasticity and intracellular force generation [94].

Similar works use the above mentioned concepts for focal adhesions simulations. Nicolas *et al.* [95] established a modeling for the growth of focal adhesions which considers thee mechanics of the focal adhesion proteins as a thin elastic layer. The authors analyzed the effect of an n applied external force, once focal adhesions have already nucleated, showing the formation of focal adhesion anisotropic growth at the direction of the applied force. This model was extended by Besser & Safran [96] in order to account for the dynamics of plaque proteins. In this sense, the authors established a phenomenological link the mechanics of the plaque and the aggregation of proteins at sites where focal adhesions are either stretched or exposed to in-plane compression. This model was further extended by Nicolas *et al.* [97] to account for the elasticity of the substrate which was considered as infinitely rigid in Besser & Safran [96]. Results for this general case show that adhesions saturate to a size that is proportional to the rigidity of the substrate with a characteristic c time that is also proportional to the rigidity [97]. Moreover, the model presented in Nicolas *et al.* [95] was additionally extended in order to a account for the elasticity of the ECM [98] since, as discussed, it plays a central role in the formation and maturation of focal adhesions. This statement was a analytically corroborated. In particular, the authors showed and increasing growth of focal adhesions with increasing matrix thickness and increasing matrix stiffness, yielding a saturation size in the case that the matrix thickness is much larger than the adhesion size. This asymptotic size of the focal adhesion can be analytically modulated by the mechanical properties of the matrix [98]. On the other hand, a simple model for circular growth of focal adhesions was presented by Gov [99].

Another different approach to model cell-biomaterial interaction was presented by Bischofs *et al.* [100]. In this work, the authors modeled the cell interacting with an elastic media (substrate) as a force dipole, which is a typical approach in the field of atomic defects. The authors presented results of a force dipole for a full space, half space and a spherical domain. On the other hand, Stéphanou *et al.* [101] modeled the growth of focal adhesions with oscillatory cell protrusions in the context of spontaneous migration. The authors considered three types of adhesion complexes, namely, the adhesion point (*A*), the focal complex (*FX*) and the focal adhesion (*FA*). According to their model, *A* can mature into a *FX* if stimulated by a moderate traction force, being then broken if the force is too strong. Moreover, *FX* can mature into *FA* if actively stimulated, that is, if the traction force from the actin filaments is beyond a given threshold force. Finally, only *FA*’s are assumed to be able to resist high stress [101]. The model was phenomenologically inspired in the experimental evidences found by Kaverina *et al.* [41]; Zaidel-Bar *et al.* [102] and Galbraith *et al.* [103]. Furthermore, Olberding *et al.* [104] developed a model available for the simulation of focal adhesion dynamics. The model is based on the hypothesis that there exist four thermodynamic forces in focal adhesion coming from different potentials, namely, the work done during the addition of a single molecular complex of a certain size, the chemical free energy change associated with the addition of a molecular complex, the elastic free energy change associated with deformation of focal adhesions and the cell membrane, and the work done on a molecular conformational change [104].

An important issue in the process of focal adhesion is the migration of binder proteins within the plaque. This phenomenon is especially relevant in focal adhesions formation during migration [15]. Freund & Lin [105] proposed a model that accounts for the diffusion of binder proteins along the membrane. This model also considers the adhesion energy of the cell as an entropic free energy of the binder distribution in the total free energy of the system. Results coming from this model simulate the rate of growth of an adhesion zone in terms on the distribution of the binders [105]. Diffusion of binders was also addressed by Wang & Gao [106]. This work shows that the uniform distribution of bonds within the membrane is intrinsically unstable with respect to perturbations in bond density distribution, mainly driven by elastic deformation energy of the system [106]. Moreover, Lin [107] presented a complete model of cell motility which considers diffusion of binder proteins across the membrane, movement of the adhesion front considering bond breaking and force generation by polymerization. Results highlight the importance of adhesion molecules in cell motility.

From a different perspective, Lin *et al.* [108] analyzed the phenomenon of adhesion as a competition between adhesive driving forces and membrane fluctuations due to Brownian motions by means of a statistical mechanics approach. Results show that membrane length must be below a critical value if bonding is to be possible at a specified level of binding energy [108].

### 3.3. Continuum Approaches

Continuum methods such as finite element (FEM) simulations allow examining mechanical experiments on cells and *in vivo* mechanical environments. Results of these simulations might be very valuable for the design of synthetic substrata for cell biology experiments and biotechnological platforms. Very few models have been developed to simulate cell-biomaterial interaction from a continuum approach. These models may be roughly classified into (i) single cell-biomaterial interaction models; (ii) cell populations-biomaterial interaction models; and (iii) indirect mechanistic models in tissue engineering.

**Figure 7:**
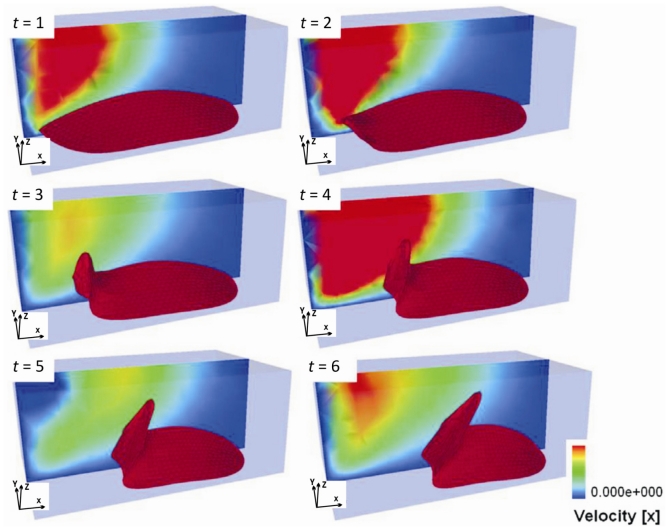
Endothelial cell detachment in the numerical framework of fluid-structure interaction. From [110].

**Figure 8:**
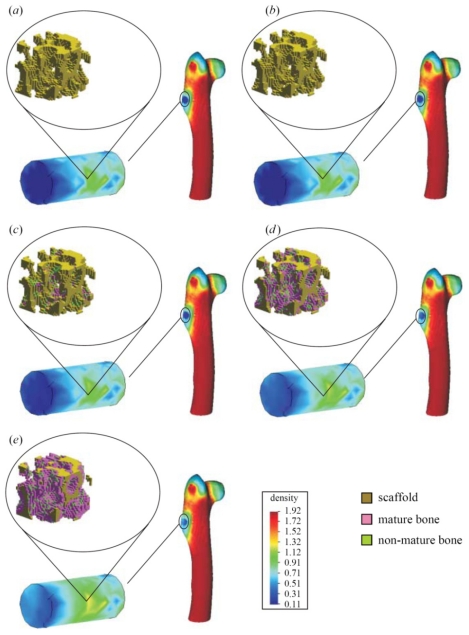
Apparent density evolution (g·cm^−3^) of a proximal femur implanted with a scaffold in the greater trochanter region. In each part, the bone organ (right), detail of the scaffold implantation (bottom left) and the bone regeneration distribution onto the scaffold microsurface of the macroscopic scaffold midpoint (top left) are shown: (**A**) 2 days; (**B**) 14 days; (**C**) 28 days; (**D**) 42 days; and (**E**) 56 days after implantation. From [139].

- *Single-cell-biomaterial interaction models:* This kind of approaches attempts to model in one hand the mechanical behavior of the CSK for a single cell, the mechanical behavior of the biomaterial on the other hand and the interaction between both by incorporating some of the characteristics of the previously discussed models. Cytoskeleton has been modeled as a continuum deformable body, as a soft glassy material [109], viscoelastic and continuum elastic [110,111], multiphasic model [112], as a gel [113], or based on the tensegrity theory [114]. So far, there is no consensus as to whether cytoskeleton behavior is closer to a fluid or to a solid, since it shows features of both. Likely, both perspectives should be accounted for in an overall global model for CSK (see Mofrad & Kamm [4] for a discussion on cell mechanics modeling). Then, the mechanical behavior of the biomaterial may be assumed under a simple viscoelastic or elastic behavior as a first approach in order to analyze its mechanical influence on cell attachment. Gracheva & Othmer [115] presented a 1D model which assumes the cell as a linear viscoelastic material interacting with the substrate through a drag (friction) coefficient. The substrate was considered as a rigid material. The main interest of that model was to analyze cell motility, so adhesion was considered only in phenomenological terms. This model was further extended to 2D in [116] accounting also for the matrix elasticity. In both models, contractile force generation and focal adhesion forces at the cell front during migration were phenomenologically modeled according to experimental evidences. In a similar model, Kuusela & Alt [117] phenomenologically modeled the force at cell-substrate interface as a drag force. However, this model considers motility of ligand and receptors adhesion proteins using diffusion-advection equations. On the other hand, Deshpande *et al.* [118] considered a contractile model for the CSK reminiscent to the Hill’s equations for muscle contraction [119]. The cell (considered as a square) is attached in the corners to four elastic elements (deflection posts) in order to analyze the effect of posts rigidity as cell contracts, as a measurement of focal adhesion forces. This model was extended in Deshpande *et al.* [120] where focal adhesion dynamics were considered in greater detail, similarly to the models discussed in the previous section, although here in a continuum framework. Kopacz *et al.* [110] simulated endothelial cell adhesion on arterial constructs under a blood flow. The cell is modeled as a viscoelastic material whereas the effect of the ECM was neglected. Focal adhesions were modeled using the classical Bell’s model [80]. Nevertheless, the focus of this work was the computational aspects of fluid-cell interaction (see Figure 7). Other works analyze cell-biomaterial interaction from another perspective rather than adhesion. For example, Sanz-Herrera *et al.* [121] investigated on the effect of substrate curvature on internal CSK forces redistribution in 3D matrices.
- *Cell-populations-biomaterial interaction models:* The main difference of this approach with the models discussed in (i) is the fact that a material point of analysis accounts for both cell and substrate, so the interaction between both cannot be easily incorporated in these models. However, cell and biomaterial mechanical behavior is modeled similar than in models presented above. Examples of these models may be found in Oster *et al.* [122]; Namy *et al.* [123]; Murray [124] and many others. Interestingly, Moreo *et al.* [125] used this approach to model the cell body as a contractile material [119] and a viscoelastic susbtrate, in an attempt to predict the experimentally observed phenomena of durotaxis and tensotaxis. Results showed an increasing concentration of cells in areas of strained and stiffer substrates.
- *Indirect mechanistic models in tissue engineering*: Some examples of continuum studies that are based on cell-center approaches are within the tissue engineering framework. These models use lattice-based methods to discretize the spatial domain into a structured grid. The random-walk model introduced by Pérez & Prendergast [126] using a lattice based approach has been successfully applied to bone tissue engineering [127–130]. Byrne *et al.* [127] and Sanz-Herrera *et al.* [131] simulated tissue growth in a scaffold to investigate various design parameters (e.g., scaffold porosity, Young’s modulus, and dissolution rate) and Khayyeri *et al.* [128] studied tissue differentiation in an *in vivo* bone chamber. The random walk model early proposed by Pérez & Prendergast [126] has been extended further to account for the vascular network formation [132] and applied to different scaffold geometries for bone tissue engineering [129,130]. Other studies within the tissue engineering field have shown how mechanical loads and fluid flow applied on the scaffolds cause different levels of mechanical stimuli on cells at a microscopic level within the samples according to the morphology of the materials [133]. Other models to analyze cell activity on scaffolds [134–140] are also available for the macroscopic design of scaffolds in terms of porosity, permeability, apparent mechanical behavior, *etc*., which should be similar to properties of the natural tissue (see Figure 8).

## 4. Perspectives

Cell-biomaterial interaction in the context of tissue engineering is a fascinating field. In one hand, engineers have the challenge of fabricating the ECM which should mimic the characteristics and functions of the natural one. This is a highly nontrivial task since the physico-mechano-chemical features of the natural ECM must be mimicked, but also it is necessary to understand the intricacies and processes of the ECM in order to replicate a functional tissue engineering product. In this framework, cell to biomaterial adhesion is critical in the first stages of the tissue engineering methodology. Adhesion is the basis and the key process which allows other important cell activities such as migration and growth. It has been discussed that the phenomenon of adhesion is highly dependent on a number of input mechanical signals translated to the cell language by the complex process of mechanotransduction. On the other hand, mathematicians, physicists and engineers have the challenge of modeling this complex process in order to predict some experimentally observed features of adhesion, which may add some light in the understanding of mechanotransduction. Moreover, advances in this field may contribute to make tissue engineering a clinical viable reality which has not been reached so far.

Different models available for the analysis of adhesion have been discussed in this paper. The different mathematical models were classified according to the scale of observation they were established, ranging from molecular models, cell-biomaterial surface models, and continuum macroscopic models which account both for the cell and the biomaterial at different levels. Molecular models have the capability to consider a great level of detail since they are posed at the protein level. Protein structure, bond stiffness and molecules interactions are some features which can be captured using the molecular approach, so this type of model may shed some light in the understanding of mechanotransduction, this being a process which takes place at the protein level. However, due to computational limitations, molecular models are far from considering the link between ligand-receptor proteins and CSK structures such as actin filaments, which is essential for a proper modeling of adhesion. In fact, these models only account for a number of binder proteins (the most relevant ones) in the simulations. Some interesting alternatives to couple molecular dynamics with a higher observation scale are coarse-graining techniques [141,142] or other bridging methods [143]. However, very few molecular dynamics models, in the framework of adhesion, have been developed so far which may be identified as an interesting future line for research. In contrast, there exists more information regarding adhesion surface modeling based on kinetics and chemomechanical models. To some extent, there exists a consensus that thermodynamical, chemical and mechanoadhesive forces drive focal adhesion. However, models are being improved to incorporate mechanical features observable at the cell body. Continuum approaches macroscopically model some of the most important phenomena of adhesion considering both the cell and the biomaterial. Due to the phenomenological nature of these models, fitting parameters and calibration of equations should be done in connection with available experimental results and also models developed at a lower observation scale. Furthermore, the cell adhesive surface of kinetic models may be coupled both with the CSK and biomaterial behaviors by means of a multiscale approach. The theoretical framework of this model has been developed in Reina-Romo & Sanz-Herrera [144] in the context of mechanics. Regarding to continuum indirect mechanistic models, an effort has been made to account, at least indirectly, for cell-biomaterial interaction. However, a great level of detail is still necessary in order to take into consideration the complexity of real tissues at different scales. Again, multiscale modeling, focus of intense research in many areas, may be a powerful technique in the future to study the complex cell-substrate interaction since this phenomenon occurs at multiple scales starting from the tissue level. Nevertheless, the statement of the problem involving more than two scales of observation seems to be computationally inaccessible in the present days. Mechanics of cell-biomaterial interaction also regards the migration of adherent cells on surfaces. This phenomenon has been treated in this paper to a lesser extent. Models in this context may be found in [145–151].

**Figure 9:**
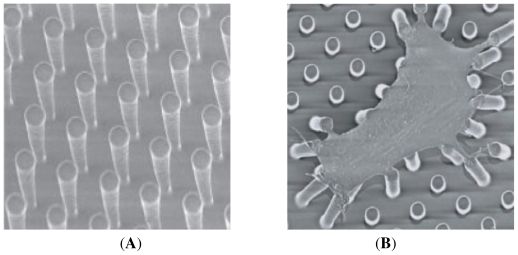
Microfabricated array of posts (**A**) and (**B**) smooth muscle cell lying on the array of posts (note on deflection due to cell contraction). From Tan *et al.* [66].

**Figure 10:**
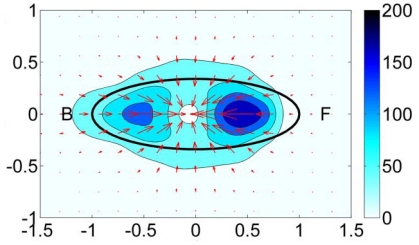
Average force field of the amoeboid form of *Dictyostelium discoideum* experimentally measured (indirectly) over the deformation of a gelatin gel substrate. The color contours indicate the magnitude of the forces in pN, and the arrows indicate their magnitude and direction. The black ellipses are least squares fits to the average shape of the cells in the cell-based reference system. From Del Alamo *et al.* [155].

The main characteristic of a model to simulate cell adhesion, for artificial substrates design in tissue engineering, is to be predictable. Then, theoretically derived models must be experimentally validated. The first report to validate the kinetics of adhesion was presented in Boulbitch *et al.* [152]. In this work, the authors measured the adhesion surface which was correlated with a diffusive model of binding/unbinding proteins. The general idea presented in this paper should be followed by modelers in order to calibrate the models and check hypothesis, by no means an easy task. Recently, high-tech methods, such as microfabrication or atomic force microscopy (AFM) among others, have been adopted in the lab to measure substrate forces exerted by the cell during adhesion. An interesting approach to measure forces exerted by the cell as it attaches to the substrate is that based on a microneedles-array substrate [66,153] (see Figure 9). Forces are then measured indirectly according to the deflection of the posts. Sagvolden *et al.* [154] measured the cell force during lamellipodial protrusion using an AFM. The authors placed the AFM cantilever tip in front of the cell body, which deflects the cantilever tip as protrusion advances, hence indirectly measuring the applied force by the cell at the front. Other experimental approaches include the measurement of substrate deformation when a cell is placed on it. Forces generated by the cell are then computed indirectly through the measured deformations once the mechanical characterization of the substrate is known [155] (see Figure 10). Results coming from these experimental setups may be used to fit model parameters used in the different models.

## 5. Conclusions

In this paper, we have discussed the fact that that adhesion is a key process in cell activity which highly depends on mechanical factors. It is in fact a complex process involving protein binders which connect the stress fibers of the CSK with the outside world, *i.e.*, the extracellular space through ECM proteins. A functional product of tissue engineering should mimic the extracellular space at the protein scale, which remains a tremendous challenge in engineering due to the complexity at this level. Then, the mechanisms of cell-biomaterial interaction in the framework of adhesion may be analogous than those that happen in the natural ECM.

A proper understanding of cell adhesion on biomaterial surfaces allows developing rational models available for artificial substrate design, which may shed some light in the task of making tissue engineering a clinical viable reality. Depending on the scale of observation, the approach is substantially different and the results should be always interpreted qualitatively after a proper model validation is conducted. In this paper, different approaches ranging from molecular simulations to continuum models have been discussed. Molecular models of adhesion need to be further developed to validate hypothesis of higher-observation-scale models and also to unravel the phenomenon of mechanotransduction. On the other hand, an effort is needed for the development of efficient coarse-grained methodologies which allow coupling the protein level with a higher-observation-scale. Results at this level may yield interesting conclusions about adhesion and biomaterials design. Therefore, there is much to be done in the context of adhesion modeling in connection to tissue engineering science. Insights in this area may mean a great advance in regenerative medicine and artificial organ fabrication fields.
